# Association Between Methemoglobin Levels and Necrotizing Enterocolitis in Preterm Infants: A Retrospective Cohort Study

**DOI:** 10.3390/children13101326

**Published:** 2026-09-30

**Authors:** Muhammet Asena, Duygu Tuncel, Leyla Şero, Nilüfer Okur

**Affiliations:** 1 Department of Pediatrics, Istanbul Medipol University, Medipol Mega University Hospital, Istanbul 34214, Turkey; 2 Clinics of Neonatology, Department of Pediatrics, Diyarbakır Gazi Yaşargil Training and Research Hospital, Diyarbakır 21090, Turkey

**Keywords:** necrotizing enterocolitis, methemoglobin, preterm, prognosis

## Abstract

**Highlights:**

**What are the main findings?**

• Methemoglobin (MetHb) levels were significantly higher in preterm infants with NEC, and higher MetHb remained independently associated with NEC after adjustment for gestational age and sex.
• MetHb alone showed poor discriminatory performance for NEC, did not distinguish Bell stage II from stage III NEC, and was not independently associated with mortality after adjustment.

**What are the implications of the main findings?**

• MetHb should not be used as a standalone diagnostic, severity, or prognostic marker for NEC because of its limited discriminatory performance.
• Any potential incremental value of MetHb within multimarker or combined clinical assessment strategies requires prospective evaluation and external validation.

**Abstract:**

**Background:** Necrotizing enterocolitis (NEC) is a leading cause of morbidity and mortality in preterm infants, and there remains a need for simple, easily measured parameters reflecting its prevalence and severity. This study tested the hypothesis that methemoglobin (MetHb), a molecule implicated in nitric oxide metabolism, is associated with the presence and severity of NEC, as well as with its complications. **Methods:** We performed a single-center, retrospective cohort study on 291 premature infants (<32 weeks’ gestational age). A total of 217 had no NEC and 74 had Bell stages II–III NEC. MetHb expressed as a percent of the total hemoglobin (% MetHb) was studied for its relationship with NEC and outcomes. Logistic regression and receiver operating characteristic analysis were performed. **Results:** % MetHb was higher among neonates who developed NEC compared to controls [2.55% (1.70–4.95) vs. 2.20% (1.70–2.80); *p* = 0.040]. For every twofold increase in % MetHb, there was an association with the development of NEC after adjustment for sex and gestational age (aOR: 1.74; 95% CI: 1.24–2.45; *p* = 0.001). Discrimination of NEC was poor, with an area under the curve of 0.580 (95% CI: 0.493–0.667); at a cutoff of ≥4.6% (exploration purposes), the test had a sensitivity of 28.4% and a specificity of 94.5%. The test failed to differentiate stage II from stage III NEC, and its association with all-cause death and death with an NEC association, after adjustment, failed to reach significance. **Conclusions:** Elevated MetHb levels were independently associated with NEC, although they lacked adequate standalone predictive and discriminatory ability; these data do not support current utilization for prediction or diagnosis, and further prospective study with standardized sampling, external validation, and a multimarker approach is needed.

## 1. Introduction

Necrotizing enterocolitis (NEC) is the leading and deadliest gastrointestinal emergency in preterm infants, with a mortality rate reaching 30% [1]. Its incidence rate inversely correlates with gestational age, and it has been diagnosed in approximately 7% of very-low-birth-weight (VLBW, <1500 g) preterm infants [1]. Recent improvements in neonatal care and the tendency to rescue extremely preterm infants have contributed to an increased population of preterm infants at risk for developing NEC.

The severity of NEC is defined based on the staging system introduced by Bell in 1978 and modified by Kliegman and Walsh, which also serves as a primary treatment guide [2]. In stage I NEC (suspected NEC) and stage II NEC (definite NEC, mild to moderate), treatment usually involves broad-spectrum antibiotics, a temporary cessation of enteral feeding, and parenteral nutrition [3]. In contrast, stage III NEC is complicated by hemodynamic instability, thrombocytopenia, disseminated intravascular coagulation, peritonitis, and pneumoperitoneum and often requires surgical intervention [3]. Moreover, mortality rates significantly increase in cases requiring surgery.

NEC is a polymicrobial, multifactorial inflammatory condition resulting from the complex interplay between host- and diet-related factors and the intestinal microbiota [3]. Disordered nitric oxide (NO) homeostasis and vascular function play a critical role in the pathogenesis of NEC. Physiologically, the neonatal circulation is biased toward vasodilation to ensure the sufficient perfusion of the energetically demanding intestine due to substantial NO production [4]. This is predominantly mediated by constitutive endothelial nitric oxide synthase (eNOS). In NEC, enhanced NO production, mainly driven by inducible nitric oxide synthase (iNOS), disrupts the intestinal homeostasis and contributes to the clinical manifestations of NEC [5]. Specifically, increased NO and neurotoxic peroxynitrite, which is formed by the reaction of NO with superoxide, suppress intestinal epithelium repair via enterocyte apoptosis and inhibition of cell proliferation and migration [6]. Meanwhile, eNOS dysfunction and consequent decreases in NO production promote vascular vasoconstriction via endothelin-1, thereby reducing mesenteric blood flow [4,7]. Altogether, these changes lead to intestinal ischemia and drive the pathogenesis of NEC.

The clinical presentation of NEC is often nonspecific, and the disease may progress rapidly. Therefore, there is continued interest in readily available parameters that may reflect the presence, severity, or biological activity of NEC and complement clinical assessment [4]. Several candidate markers, including intestinal alkaline phosphatase, intestinal fatty acid-binding protein (I-FABP), and calprotectin, have been investigated in relation to NEC diagnosis, severity, and outcomes [8,9,10].

Methemoglobin (MetHb) has also been investigated in the context of altered nitric oxide metabolism, although data regarding its relationship with NEC remain limited. MetHb is a hemoglobin derivative formed by oxidation of ferrous iron to ferric iron and has been proposed as a marker of increased nitric oxide production in critically ill patients [11]. Because nitric oxide can contribute to MetHb formation, circulating MetHb has been proposed as an accessible surrogate of altered NO bioactivity in critically ill patients [11,12]. Increased MetHb levels have also been reported in patients with sepsis and septic shock [11,12] and have been associated with illness severity and adverse outcomes [11,13]. Given the important role of altered nitric oxide homeostasis in NEC pathophysiology, MetHb may reflect biological processes associated with NEC. However, its association with the presence and severity of NEC and with clinical outcomes has not been sufficiently characterized.

Therefore, this study aimed to evaluate the association between MetHb levels and the presence of Bell stages II–III NEC in preterm infants born before 32 weeks of gestation. Secondary objectives were to assess the associations of MetHb with NEC severity and mortality and to evaluate its discriminatory performance for these outcomes.

## 2. Materials and Methods

### 2.1. Study Design and Setting

This was a single-center, retrospective cohort study performed in a tertiary neonatal care unit. Medical records of preterm infants admitted to the Neonatal Intensive Care Unit (NICU) who met the inclusion criteria were reviewed retrospectively. The study followed the ethical principles of the Declaration of Helsinki, and reporting was guided by the Strengthening the Reporting of Observational Studies in Epidemiology (STROBE) recommendations. The study was approved by the Diyarbakır Gazi Yaşargil Training and Research Hospital Clinical Ethics Committee (2 October 2024; approval no. 209). Because of the retrospective design and use of anonymized data, the requirement for individual informed consent was waived by the ethics committee.

### 2.2. Study Population and Definition of Groups

Preterm infants born at <32 weeks’ gestation and admitted to the NICU at our center between 1 June 2023 and 1 August 2024 were eligible for inclusion. Our hospital is a tertiary referral center serving a catchment population of approximately 10 million people, and high-risk preterm deliveries from the surrounding region are routinely referred to our center. Owing to the high patient volume, shortages in available NICU incubators may occasionally occur; in such circumstances, relatively more stable infants may be transferred shortly after birth to other neonatal centers within the same city.

During the study period, 379 infants born at <32 weeks’ gestation were screened. Of these, 48 were transferred to external centers immediately after birth because an appropriate NICU incubator was unavailable, 18 were excluded because of major congenital anomalies or gastrointestinal obstructions, and 22 were excluded because they died within the first 72 h of life. Early deaths were excluded to reduce marked differences in the opportunity to develop NEC before outcome classification.

Details of the cohort selection are depicted in Figure 1.

This selection resulted in a final study cohort of 291 babies.

Infants with major gastrointestinal tract congenital abnormalities, significant congenital anomalies expected to affect study outcomes, congenital methemoglobinemia, or other genetic hemoglobin disorders were excluded. Infants with insufficient clinical or laboratory data to determine the main study parameter or establish NEC status were also excluded. Neonates with recurrent NEC were included only once, and the highest Bell stage reached during hospitalization was used for classification.

NEC was diagnosed according to the Modified Bell criteria, and only Bell stage II–III disease was classified as definite NEC. Infants with Bell stage I NEC were included in the no-NEC group because suspected NEC at this stage cannot always be reliably distinguished from late-onset neonatal sepsis on clinical grounds. In our unit, these infants are generally managed conservatively with the temporary interruption of enteral feeding for at least two days and, when clinically indicated, antibiotic therapy.

For the analyses, infants were classified into the no-NEC group or NEC group. The NEC group comprised infants with Bell stage II or III disease; when progression occurred, the highest stage reached was used for classification. The final cohort included 217 infants without definite NEC and 74 infants with NEC, of whom 49 had stage II disease and 25 had stage III disease.

### 2.3. Methemoglobin Measurement

For the measurements, MetHb was the variable of interest and was extracted from blood gas analysis reports, expressed as a percentage of the total hemoglobin. The MetHb values in infants with NEC were those prior to the diagnosis of NEC.

Where more than one measurement was taken prior to the diagnosis of NEC, the highest absolute MetHb value was used for analysis, regardless of the interval since diagnosis.

Only one MetHb value was included per infant. For the no-NEC infants, the highest MetHb value recorded at any point during the course of the hospital stay was included. Blood gas analyses are routinely performed in extremely preterm and very-low-birth-weight infants and are often taken more regularly during episodes of clinical deterioration, including during sepsis, when hemodyamically significant patent ductus arteriosus is present, where mechanical ventilation is used or with suspected or confirmed NEC. Due to the retrospective nature of the study, the postnatal age of blood gases taken, the number and frequency of blood gas measurements taken in each infant, and the duration of the interval between the highest recorded MetHb measurement and NEC diagnosis were not reliably recorded, precluding standardization of the sampling frequency or comparison of sampling intervals between cases with NEC and no NEC. The other requested blood gas measurements were not obtained specifically for the purposes of this study. The clinical parameters that may influence MetHb levels were obtained from clinical records.

### 2.4. Clinical and Demographic Variables

Gestational age, birth weight, maternal age, 1 and 5 min Apgar scores, sex, mode of delivery, and antenatal steroid exposure were retrieved from clinical records. Coding for sex and mode of delivery was verified against the original records. NEC diagnosis and the Modified Bell stage were recorded as the principal clinical variables. Anemia was defined according to gestational age and postnatal age and its prevalence was compared between infants with and without NEC. In-hospital mortality and NEC-related mortality were also recorded. Two mortality outcomes were defined. All-cause in-hospital mortality included death from any cause during hospitalization. NEC-related mortality was defined as death attributable to NEC. A total of 109 in-hospital deaths occurred in the main analysis cohort, including 15 NEC-related deaths among infants with NEC. Sepsis was defined by isolation of a microorganism from blood culture.

### 2.5. Study Outcomes

The primary outcome was the association between MetHb levels and the presence of NEC at Bell stages II–III. Secondary endpoints included the association between MetHb levels and all-cause in-hospital mortality, as well as discrimination for NEC. Analyses of NEC-related mortality and stage III versus stage II NEC were considered exploratory because of the very small number of events. ROC analysis assessed the standalone discriminative ability of MetHb.

### 2.6. Statistical Analysis

Continuous variables were summarized as mean ± standard deviation (SD) or median with interquartile range (IQR), according to their distribution. Categorical variables are presented as numbers and percentages. Comparisons between two independent groups were performed using the independent-samples *t*-test or Mann–Whitney U test for continuous variables and the chi-square test or Fisher’s exact test for categorical variables, as appropriate.

Anemia was defined according to gestational age and postnatal age and was analyzed as a categorical variable. Its prevalence was compared between the NEC and no-NEC groups using the chi-square test or Fisher’s exact test, as appropriate.

Because MetHb showed a right-skewed distribution, MetHb values were log2-transformed for the principal regression analyses. Accordingly, odds ratios (ORs) for MetHb represent the change in odds associated with each twofold increase in MetHb.

The primary analysis evaluated the association between MetHb and the presence of Bell stages II–III NEC using multivariable logistic regression adjusted for gestational age and sex. Gestational age was selected because of its established association with NEC risk and its confounding effect on the MetHb-NEC relationship, while sex was included as a prespecified demographic covariate. Because birth weight is closely related to gestational age and represents an alternative marker of neonatal maturity and vulnerability, a sensitivity analysis was performed in which birth weight replaced gestational age while sex remained in the model.

As an additional exploratory analysis, gestational age, birth weight, and untransformed MetHb were entered simultaneously into a multivariable logistic regression model to assess whether the association between MetHb and NEC persisted when both major measures of prematurity were considered together. In this exploratory model, the MetHb effect was expressed per 1-percentage-point increase. Multicollinearity was assessed using variance inflation factors (VIFs). This model was considered supportive rather than primary.

All-cause in-hospital mortality was evaluated as a secondary outcome using the same log2-transformed MetHb modeling strategy. Analyses of NEC-related mortality and stage III NEC were considered exploratory because of the limited number of events and were interpreted cautiously; nonsignificant findings were not considered definitive evidence of absence of association.

ROC curve analyses were used to evaluate the discriminatory performance of MetHb for NEC, the all-cause mortality, and stage III versus stage II NEC. Areas under the curve (AUCs) with 95% confidence intervals (CIs) were reported. Exploratory cutoff values were identified using the Youden index, with the corresponding sensitivity and specificity; positive and negative predictive values were additionally reported for the NEC analysis.

Potential confounding variables were further explored by assessing their influence on the MetHb regression coefficient. These analyses were considered exploratory and were used to support covariate selection and interpretation rather than to define additional primary models.

All statistical tests were two-sided, and *p* < 0.05 was considered statistically significant. Statistical analyses were performed using IBM SPSS Statistics version 26.0 (IBM Corp., Armonk, NY, USA).

## 3. Results

A total of 291 preterm infants were included in this study; 217 (74.6%) were classified as no-NEC and 74 (25.4%) had Bell stages II–III NEC. Among infants with NEC, 49 had stage II disease and 25 had stage III disease. In the entire cohort, 109 in-hospital deaths occurred, including 15 NEC-related deaths among infants with NEC. Among infants who developed NEC, the median postnatal age at diagnosis was 8 days (IQR, 6–12 days). Among infants who did not develop NEC, 81 died during hospitalization; among those with available data on postnatal age at death, the median age at death was 6 days (IQR, 4–29 days). The demographic characteristics are presented as shown in Table 1.

**Table 1:** Demographic and clinical characteristics of preterm infants.

Variable	No NEC (*n* = 217)	NEC (*n* = 74)	*p*
Gestational age, weeks	28.0 (27.0–30.0)	27.0 (26.0–28.0)	<0.001
Birth weight, g	1175.0 (875.0–1406.2)	900.0 (800.0–1100.0)	<0.001
Maternal age, years	27.0 (23.0–33.0)	28.0 (25.0–34.0)	0.225
1 min Apgar score	5.0 (3.0–6.0)	5.0 (4.0–6.0)	0.411
5 min Apgar score	6.0 (5.0–7.0)	7.0 (6.0–7.0)	0.251
Sex—male	110 (50.7%)	37 (50.0%)	1.000
Mode of delivery—NVD	30 (13.8%)	10 (13.5%)	1.000
* Antenatal steroids—none	90 (41.7%)	37 (50.0%)	0.460
Antenatal steroids—single dose	34 (15.7%)	10 (13.5%)	
Antenatal steroids—complete course	92 (42.6%)	27 (36.5%)	
Anemia, *n* (%)	76 (35.0%)	31 (41.9%)	0.29

Data are presented as median (IQR) or *n* (%), according to distribution. The Mann–Whitney U test or independent-samples *t*-test was used for continuous variables, and the chi-square test or Fisher’s exact test was used for categorical variables. Coding for sex and mode of delivery was verified against the original records. NEC: necrotizing enterocolitis; NVD: normal vaginal delivery. * One infant in the no-NEC group was excluded from the antenatal steroid analysis because antenatal steroid exposure was uncertain.

Infants with NEC had significantly lower gestational ages and birth weights than infants without NEC (both *p* < 0.001). Maternal age, 1 and 5 min Apgar scores, sex, mode of delivery, and antenatal steroid exposure did not differ significantly between groups. Anemia was present in 76 (35.0%) infants in the no-NEC group and in 31 (41.9%) infants in the NEC group, with no statistically significant difference between the groups (*p* = 0.29).

MetHb levels were higher in infants with NEC than in infants without NEC [2.55% (1.70–4.95) vs. 2.20% (1.70–2.80); *p* = 0.040] (Table 2). In univariable analysis, each twofold increase in MetHb was associated with higher odds of NEC (OR: 1.93; 95% CI: 1.38–2.70; *p* < 0.001). This association remained significant in the primary model adjusted for gestational age and sex (aOR: 1.74; 95% CI: 1.24–2.45; *p* = 0.001). The result was similar in the prespecified sensitivity model in which birth weight replaced gestational age (aOR: 1.76; 95% CI: 1.26–2.46; *p* < 0.001).

MetHb levels were higher in non-survivors than in survivors [2.70% (1.80–3.70) vs. 1.95% (1.70–2.60); *p* < 0.001] (Table 3). MetHb was associated with all-cause mortality in univariable analysis (OR: 1.52; 95% CI: 1.13–2.06; *p* = 0.006), but the association was attenuated and was no longer statistically significant after adjustment for gestational age and sex (aOR: 1.32; 95% CI: 0.97–1.81; *p* = 0.081). The sensitivity model adjusted for birth weight and sex yielded a similar result (aOR: 1.29; 95% CI: 0.94–1.77; *p* = 0.114).

In this exploratory analysis, the median MetHb level was numerically higher among infants with NEC-related death than among those without NEC-related death [4.40% (1.75–7.20) vs. 2.50% (1.70–3.80); *p* = 0.212] (Table 4). No statistically significant association was observed in either the univariable or gestational age- and sex-adjusted logistic regression analysis (*p* = 0.288 and *p* = 0.371, respectively). Given the limited number of NEC-related deaths, these estimates were considered exploratory and should be interpreted cautiously.

MetHb levels were similar in infants with stage II and stage III NEC [2.60% (1.70–4.60) vs. 2.50% (1.50–5.70); *p* = 0.977] (Table 5). The exploratory logistic regression model adjusted for gestational age and sex was not statistically significant (aOR: 1.13; 95% CI: 0.75–1.70; *p* = 0.568), and the ROC AUC was 0.502. Because only 25 infants had stage III NEC, these findings were considered exploratory rather than definitive evidence of absence of association.

When MetHb alone was evaluated for discrimination of NEC, the AUC was 0.580 (95% CI: 0.493–0.667) (Table 6). The 95% CI included 0.50, indicating a poor discriminatory performance that was not statistically distinguishable from chance. The Youden index identified an exploratory cutoff of MetHb ≥ 4.6%, with 28.4% sensitivity, 94.5% specificity, 63.6% positive predictive value, and 79.5% negative predictive value. This cutoff was derived from the same cohort and was not independently validated.

In the exploratory model that simultaneously included gestational age, birth weight, and untransformed MetHb, higher MetHb remained associated with NEC (aOR: 1.32 per 1-percentage-point increase; 95% CI: 1.15–1.53; *p* < 0.001) (Table 7). Thus, each 1-percentage-point increase in MetHb corresponded to 32% higher odds of NEC, not a 32% increase in probability. Gestational age showed a borderline inverse association (aOR: 0.84; *p* = 0.054), whereas birth weight was not independently significant (aOR: 0.94; *p* = 0.283). The model AUC was 0.720. VIF values were 2.07 for gestational age, 2.06 for birth weight, and 1.02 for MetHb, indicating no problematic multicollinearity. This model was considered supportive and exploratory rather than the primary analysis.

Across endpoints, MetHb showed poor discrimination for NEC and only limited discrimination for all-cause mortality (Table 8). Although the exploratory AUC for all-cause mortality was 0.648 with a cutoff of ≥2.2%, MetHb was not independently associated with mortality in the adjusted model. The AUC for stage II versus stage III NEC was 0.502, and no clinically useful cutoff was identified.

In the exploratory confounding assessment, gestational age and birth weight produced the largest changes in the MetHb coefficient, particularly in the mortality model (Table 9). Sex, maternal age, the mode of delivery, and the 1 min Apgar score did not materially alter the MetHb-NEC association, whereas the 5 min Apgar score showed a modest effect in the mortality model. Overall, the primary and sensitivity analyses consistently showed an association between higher MetHb and the presence of NEC, while the standalone discriminatory performance of MetHb remained poor.

## 4. Discussion

The primary result of this study was an association between the presence of NEC and higher levels of MetHb, yet MetHb’s discriminatory performance was poor on its own. A twofold increase in MetHb levels corresponded to a 74% higher risk of NEC (OR: 1.74; 95% CI: 1.24–2.45) in the primary model controlling for gestational age and sex. The AUC for discrimination of NEC based on MetHb alone was only 0.580 (95% CI: 0.493–0.667), with the 95% confidence interval including 0.50. This indicates that discrimination by MetHb alone was poor and was not significantly different than random chance. The exploratory cutoff of 4.6% MetHb identified using the Youden index had a sensitivity of 28.4% and a specificity of 94.5%. Because the cutoff was derived from and applied to the same study population and was not externally validated, this should not be interpreted as a clinically validated diagnostic cutoff.

Infants with NEC in our cohort had significantly lower gestational ages and birth weights than those without NEC. This finding is consistent with the well-established susceptibility of very preterm and very-low-birth-weight infants to NEC [1,14]. Prematurity is considered a central component of NEC pathogenesis, reflecting the immaturity of intestinal barrier function, immune responses, microvascular regulation, and host–microbiome interactions [14]. Prematurity leads to the development of immaturity in microvascular regulation, intestinal mucosal barriers, host immunity, and colonization by microorganisms, and all of these can play a role in NEC vulnerability [14]. The premise, according to Hunter et al., is that prematurity is pivotal to the pathogenesis of NEC [14].

There was no difference between the two groups in terms of maternal age at the time of delivery, sex, number of APGAR scores <7, and exposure rate to antenatal corticosteroids or prevalence of anemia. The crucial finding of this study statistically was that MetHb level was independently associated with the presence of NEC on both the primary adjusted analysis on the basis of sex and gestational age and the sensitivity-adjusted analysis on the basis of sex and birth weight. As a whole, each 1-percentage-point increase in untransformed MetHb was associated with 32% increased odds of NEC in other additional exploratory analysis models (aOR: 1.32; 95% CI: 1.15–1.53), although this reflects an increase in odds and not probability.

Epithelial damage has been attributed to elevated levels of NO produced in an inducible nitric oxide synthase-dependent manner, whereas impaired endothelial NO signaling may lead to impaired vasodilation, a shift toward vasoconstriction, reduced mesenteric blood flow, and intestinal ischemia [4,5,6]. Since MetHb is formed during an oxidation reaction in NO metabolism, a finding of increased MetHb could be indicative of a general systemic or intestinal inflammatory and oxidation process.

Increased levels of MetHb have also been identified in critically ill patients with sepsis where NO metabolism pathways seem to become upregulated in an inducible nitric oxide synthase-mediated pathway [11,12,13]. These findings help to strengthen the rationale for MetHb levels being an unspecific correlate of an inflammatory process and altered NO pathways. The relationship in our patients may thus just reflect underlying inflammatory processes and circulatory abnormalities during NEC. However, the retrospective design does not exclude the possibility that increased levels may either be one component of the NEC process, or may merely occur after signs have already presented, or be a marker for another simultaneous finding, such as the more aggressive overall clinical presentation seen within this subpopulation of patients.

In terms of the significance of the low discriminatory ability of MetHb, the exploratory cutoff (4.6%) demonstrated high relative specificity (94.5%) but relatively low sensitivity (28.4%) and thus limited clinical utility in NEC screening. We also note that this cutoff was obtained using the same sample cohort and has yet to undergo validation in an external study population. Thus, at this time, MetHb should not be used in isolation for screening as a diagnostic or rule-out test for NEC. Other putative biomarkers, such as I-FABP, have demonstrated higher discriminatory estimates in prior research, though clinical utility remains incomplete due to heterogeneity and limited clinical use [15].

MetHb offers an advantage of readily available information on standard blood gas analysis, which does not require further venipuncture of the patient. Our study attempted to identify MetHb on its own, without attempting to prove its role as adjunctive to or replacing a multi-biomarker panel or overall clinical model. This would need to be established as a hypothesis in future research and was not confirmed in the current study. Large prospectively designed trials with defined windows and a standardized, clinically comparable control event period and external validation would thus be required. In this cohort, MetHb was not discriminatory between Bell stage II and stage III NEC (AUC 0.502). Neither the adjusted nor unadjusted exploratory analysis demonstrated a statistically significant association with stage III disease, although only 25 subjects suffered stage III NEC and thus estimates were imprecise. This was best interpreted as evidence of a lack of demonstrated discrimination within the cohort and not definitive of a lack of association.

Several other markers of severity and NEC outcomes have been studied, such as serum or fecal calprotectin (an indicator of surgical requirement and death in some studies) [10]; I-FABP (which may be elevated in intestinal injury, but clinical utility is unknown) [15]; and intestinal alkaline phosphatase as a marker of intestinal injury and NEC [8].

MetHb levels were higher in those who did not survive at univariate analysis but were attenuated following adjustment for gestational age and sex and failed to reach statistical significance in the adjusted model. A similar finding was noted in the analysis when birth weight replaced gestational age. These findings suggest that the crude association is likely due in part to prematurity and associated vulnerability, which are well-established risk factors for adversity and death in preterm infants, as well as for NEC mortality [16]. Therefore, results for mortality should not be interpreted as evidence of an independent association between MetHb levels and mortality. For deaths related specifically to NEC, no statistically significant association was seen with levels of MetHb.

However, there were 15 subject deaths specifically related to NEC, which was too few events to be a meaningful endpoint in even a prespecified exploratory analysis, so this absence of significance was considered inadequate to rule out association, and a study with a much larger cohort would be needed.

There are a number of limitations that should be noted in this study. First, as a single-center, retrospective study, conclusions about causation or the validity of any finding to other sites are hampered, and a residual effect of uncontrolled confounding cannot be ruled out. Second, blood gases were obtained at the physician’s discretion, and the exact frequency and number of Metrohb tests per subject were not maintained; perhaps the presence of NEC was itself an indicator to obtain blood gas (which is the basis for measurement opportunity bias or ascertainment bias). This sensitivity analysis was not possible due to missing data on the precise postnatal timing of death in a subgroup of infants; thus, the implications of time at risk for NEC in study survivors and those who died in this analysis should be considered. Third, the windows from which MetHb were sampled were not comparable across groups. MetHb assessment in infants with NEC was limited to measurements taken prior to NEC diagnosis, but the maximum MetHb while in the hospital was taken in infants without NEC. Furthermore, we did not have systematic information on individual MetHb measurement time points, including postnatal age and time of measurement. As such, we were unable to perform a postnatal age-matched index-time sensitivity analysis or calculate the time between each MetHb measurement and NEC diagnosis. Sampling to time of blood gases was more frequent in response to clinical deterioration, which might have created more opportunities to measure higher MetHb levels. This constitutes an ascertainment bias and makes interpretation of MetHb as a predictor more difficult.

Fourth, there is no external validation for the chosen ROC cutoffs, as they were determined and measured in the same population and therefore have their own specific values too and may not be generalizable.

## 5. Conclusions

Higher MetHb levels were independently associated with the presence of Bell stages II–III NEC in preterm infants. However, MetHb alone showed a poor discriminatory performance for NEC and did not reliably distinguish between stage II and stage III disease. The present findings therefore do not support the use of MetHb as a standalone diagnostic, severity, or prognostic marker for NEC. Given the retrospective, single-center design and non-equivalent sampling windows, these results should be interpreted as evidence of association rather than predictive or diagnostic utility. Future prospective studies with standardized pre-diagnostic sampling, comparable control time points, and external validation are required to determine whether MetHb provides incremental information when combined with other clinical or laboratory parameters.

## Figures and Tables

**Figure 1:**
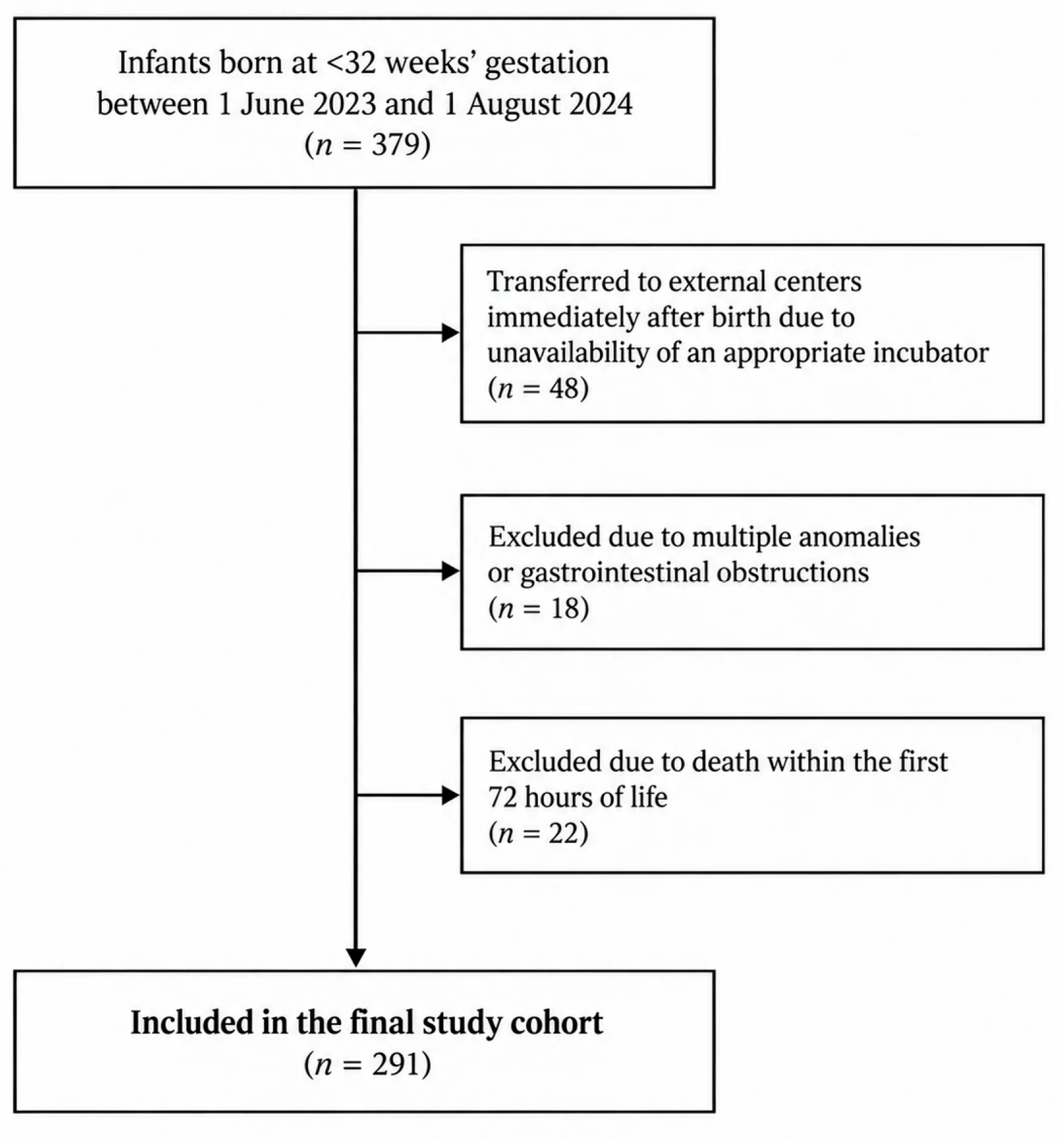
Flow diagram of study cohort selection.

**Table 2:** Relationship between MetHb level and presence of NEC.

Analysis	No NEC	NEC	Effect Estimate	95% CI	*p*
MetHb, %, median (IQR)	2.20 (1.70–2.80)	2.55 (1.70–4.95)	-	-	0.040
Univariable logistic regression	-	-	OR 1.93	1.38–2.70	<0.001
Primary adjusted model: gestational age + sex	-	-	aOR 1.74	1.24–2.45	0.001
Sensitivity model: birth weight + sex	-	-	aOR 1.76	1.26–2.46	<0.001

Regression estimates are expressed per twofold increase in MetHb (%) because log2-transformed MetHb was used. OR: odds ratio; aOR: adjusted odds ratio; CI: confidence interval.

**Table 3:** Relationship between MetHb level and all-cause in-hospital mortality.

Analysis	Survivors	Non-Survivors	Effect Estimate	95% CI	*p*
MetHb, %, median (IQR)	1.95 (1.70–2.60)	2.70 (1.80–3.70)	-	-	<0.001
Univariable logistic regression	-	-	OR 1.52	1.13–2.06	0.006
Adjusted model: gestational age + sex	-	-	aOR 1.32	0.97–1.81	0.081
Sensitivity model: birth weight + sex	-	-	aOR 1.29	0.94–1.77	0.114

Regression estimates are expressed per twofold increase in MetHb (%).

**Table 4:** Exploratory analysis of MetHb and NEC-related mortality among infants with NEC.

Analysis	No-NEC-Related Death	NEC-Related Death	Effect Estimate	95% CI	*p*
MetHb, %, median (IQR)	2.50 (1.70–3.80)	4.40 (1.75–7.20)	-	-	0.212
Univariable logistic regression	-	-	OR 1.28	0.81–2.03	0.288
Adjusted model: gestational age + sex	-	-	aOR 1.25	0.77–2.02	0.371

**Table 5:** Exploratory analysis of MetHb for differentiating Bell stage II and stage III NEC.

Measure	Stage II NEC (*n* = 49)	Stage III NEC (*n* = 25)	Result
MetHb, %, median (IQR)	2.60 (1.70–4.60)	2.50 (1.50–5.70)	*p* = 0.977
Adjusted model: gestational age + sex	-	-	aOR: 1.13; 95% CI: 0.75–1.70; *p* = 0.568
ROC AUC	-	-	0.502

**Table 6:** ROC analysis of MetHb for discrimination of NEC.

ROC Parameter	Result
AUC	0.580
95% CI	0.493–0.667
Exploratory cutoff (Youden index)	MetHb ≥ 4.6%
Sensitivity	28.4%
Specificity	94.5%
Positive predictive value (PPV)	63.6%
Negative predictive value (NPV)	79.5%

**Table 7:** Exploratory multivariable logistic regression model for NEC with simultaneous inclusion of gestational age, birth weight, and untransformed MetHb.

Variable	Adjusted OR	95% CI	*p*
Gestational age, +1 week	0.84	0.70–1.00	0.054
Birth weight, +100 g	0.94	0.83–1.05	0.283
MetHb, +1 percentage point	1.32	1.15–1.53	<0.001

**Table 8:** Endpoint-specific ROC thresholds and discrimination performance for MetHb.

Endpoint	AUC (95% CI)	Cutoff	Sensitivity	Specificity
NEC	0.580 (0.493–0.667)	≥4.6%	28.4%	94.5%
All-cause mortality	0.648 (0.579–0.716)	≥2.2%	71.6%	57.1%
Stage II vs. stage III NEC	0.502	No clinically useful cutoff	-	-

**Table 9:** Exploratory evaluation of potential confounders in MetHb–endpoint relationships.

Potential Confounder	Change in MetHb Coefficient: NEC Model	Change in MetHb Coefficient: Mortality Model
Gestational age	Approximately 15.7%	Approximately 33.4%
Birth weight	Approximately 14.3%	Approximately 38.1%
5 min Apgar score	Not substantial	Approximately 13.4%
Sex	Not substantial	Not substantial
Maternal age	Not substantial	Not substantial
Mode of delivery	Not substantial	Not substantial
1 min Apgar score	Not substantial	Not substantial

## Data Availability

Datasets analyzed during this study are available from the corresponding author upon reasonable request. The data are not publicly available in order to protect the privacy and confidentiality of the patients’ personal information.

## Abbreviations

The following abbreviations are used in this manuscript:

aOR	Adjusted Odds Ratio
AUC	Area Under The Curve
CI	Confidence Interval
C/S	Cesarean Section
eNOS	Endothelial Nitric Oxide Synthase
IQR	Interquartile Range
MetHb	Methemoglobin
NEC	Necrotizing Enterocolitis
NICU	Neonatal Intensive Care Unit
NO	Nitric Oxide
NPV	Negative Predictive Value
NVD	Normal Vaginal Delivery
OR	Odds Ratio
PPV	Positive Predictive Value
ROC	Receiver Operating Characteristic
SD	Standard Deviation
STROBE	Strengthening the Reporting of Observational Studies in Epidemiology
VIF	Variance Inflation Factor;
VLBW	Very Low Birth Weight
